# Automatized lung disease quantification in patients with COVID-19 as a predictive tool to assess hospitalization severity

**DOI:** 10.3389/fmed.2022.930055

**Published:** 2022-08-29

**Authors:** Julien Guiot, Nathalie Maes, Marie Winandy, Monique Henket, Benoit Ernst, Marie Thys, Anne-Noelle Frix, Philippe Morimont, Anne-Françoise Rousseau, Perrine Canivet, Renaud Louis, Benoît Misset, Paul Meunier, Jean-Paul Charbonnier, Bernard Lambermont

**Affiliations:** ^1^Respiratory Department, University Hospital of Liège, Liège, Belgium; ^2^Biostatistics and Medico-Economic Information Department, University Hospital of Liège, Liège, Belgium; ^3^Intensive Care Department, University Hospital of Liège, Liège, Belgium; ^4^Department of Radiology, University Hospital of Liège, Liège, Belgium; ^5^Thirona B.v., Nijmegen, Netherlands

**Keywords:** SARS-CoV-2, CT scan analysis, artificial intelligence, mechanical ventilation risk, severity of hospital stay prediction, COVID-19, in-hospital death, ICU length of stay

## Abstract

The pandemic of COVID-19 led to a dramatic situation in hospitals, where staff had to deal with a huge number of patients in respiratory distress. To alleviate the workload of radiologists, we implemented an artificial intelligence (AI) - based analysis named CACOVID-CT, to automatically assess disease severity on chest CT scans obtained from those patients. We retrospectively studied CT scans obtained from 476 patients admitted at the University Hospital of Liege with a COVID-19 disease. We quantified the percentage of COVID-19 affected lung area (% AA) and the CT severity score (total CT-SS). These quantitative measurements were used to investigate the overall prognosis and patient outcome: hospital length of stay (LOS), ICU admission, ICU LOS, mechanical ventilation, and in-hospital death. Both CT-SS and % AA were highly correlated with the hospital LOS, the risk of ICU admission, the risk of mechanical ventilation and the risk of in-hospital death. Thus, CAD4COVID-CT analysis proved to be a useful tool in detecting patients with higher hospitalization severity risk. It will help for management of the patients flow. The software measured the extent of lung damage with great efficiency, thus relieving the workload of radiologists.

## Introduction

The rapid outbreak of coronavirus disease 2019 (COVID-19), originating from severe acute respiratory syndrome coronavirus 2 (SARS-CoV-2) infection, has become a public health emergency of international concern (1). During the first wave, a high proportion of infected patients required hospitalization in the intensive care unit (ICU) (2).

Since the onset of the COVID-19 pandemic, chest CT imaging has been widely used to help clinicians in the identification of patients infected with COVID-19 (3, 4). CT scans capture imaging features from the lung associated with COVID-19 since the earliest stages of the disease. CT scan could, thus, serve as an efficient and effective way to diagnose, and possibly prognosis patients with COVID-19 admitted to the hospital.

Visual assessment of disease severity by CT scoring usually includes ground-glass opacity, consolidation, air bronchogram, crazy paving, nodular opacities, and pleural effusion (5). This method is time-consuming, requires experienced radiologists, and could be error-prone when the workload is heavy. CT scoring methods may produce different severity levels, while some effort has been made to get a common lexicon and scoring (6, 7).

However, visual scoring of CT scans is known to be susceptible to high inter-reader variability (8, 9) and allows only for qualitative or semiquantitative assessment of the parenchymal involvement of the disease, even if a major effort has been made to standardize the description of CTs used for diagnostic purposes (6). Furthermore, in a context of a high burden on healthcare institutes during the COVID-19 pandemic, visual scoring of many CT scans could be highly challenging for radiologists (10).

The Dutch Radiological Society developed the coronavirus disease 2019 (COVID-19) Reporting and Data System (CO-RADS) as a categorical assessment scheme for pulmonary involvement of COVID-19 at unenhanced chest CT (9, 11–13). This method performs well in predicting COVID-19 in patients with moderate-to-severe symptoms and has a substantial interobserver agreement. Thus, it is helpful in COVID-19 diagnosis and the evaluation of disease severity and prognosis (14).

The CO-RADS artificial intelligence (AI) system consists of three deep learning algorithms that automatically segment the five pulmonary lobes, assign the CO-RADS score for the suspicion of COVID-19, and assign a CT severity score for the degree of parenchymal involvement per lobe. This algorithm proved to be in accordance with diagnoses obtained from experienced radiologists (10), exhibiting a high performance for diagnosis and disease prognosis.

Artificial intelligence-based models can aid the radiologist in assessing CT scans, providing rapid and quantitative information on disease-related parenchymal involvement (15). These models may help to provide precise and reproducible quantitative information on lung parenchyma affected by COVID-19, while relieving some of the burden on healthcare professionals (9, 11–13). CT scan analysis with deep learning methods even allowed for the diagnosis of COVID-19 disease earlier than reverse transcriptase-PCR (RT-PCR) (8).

Besides the chest imaging effectiveness in COVID-19 diagnosis, multiple prognosis models have been developed, without making it possible to establish a strong predictive model of the clinical evolution (3).

Computed tomography scores have been combined with other clinical or biological parameters, either directly related to lung function, or inflammation or infection [C-reactive protein (CRP), D-dimer, alkaline phosphatase, etc.,] (16–19) with a good correlation with disease severity and death risk. However, we must note a lack of consistency, as the considered laboratory data differ between studies.

One of the key questions when caring for hospitalized patients with COVID-19 infection remains to determine the risk of deterioration leading to ICU admission. It is even more important to predict the need for mechanical ventilation, given the limited number of ventilators and the need for specialized staff to monitor closely these patients.

In this study, CT quantification of COVID-19-related parenchymal abnormalities was performed using CAD4COVID-CT (Thirona, Nijmegen, Netherlands). CAD4COVID-CT is a CE (0344) class IIa certified AI-based software package that automatically quantifies the lobar extent of COVID-19 using state of the art deep learning techniques. The algorithm provides a quantitative assessment of the categorical CT severity score (CT-SS) such as the CO-RADS severity scoring system, and, in addition, quantifies the volume percentage of COVID-19-related affected areas (% AA) on the lobar level.

The main goal of this retrospective study was to explore the prognostic value of CAD4COVID-CT severity scores on hospitalization severity indicators of patients with COVID-19.

## Materials and methods

### Study design and participants

During the COVID-19 pandemic, our hospital expanded its total ICU capacity from 58 to 68 beds during wave 1 (W1) (from 10 March to 22 June 2020) and 71 beds during wave 2 (W2) (from 31 August to 12 October 2020), with 10–12 beds dedicated to non-COVID-19 critically ill patients. In the whole hospital, 196 beds were dedicated to patients with COVID-19 during the two waves, out of a total of 878 beds.

All the adult patients admitted to the University Hospital of Liege for acute respiratory failure related to SARS-CoV-2 pneumonia between 13 March 2020 and 18 April 2021 were included, if they had undergone a chest CT scan at most 1 day before or after hospital admission. Patients primarily hospitalized for scheduled or urgent surgery with positive SARS-CoV-2 PCR were excluded.

Patients were diagnosed with a positive PCR for SARS-CoV-2 in nasal swabs or other respiratory samples during the 5 days of their admission to the hospital or the 14 days before admission. When performed in our hospital, the detection of SARS-CoV-2 was performed by reverse transcription PCR using the Cobas SARS-CoV-2 Assay (Roche, Switzerland) for the detection of the *ORF1ab* and *E* genes. The results were reported as cycle thresholds to have a semiquantitative measurement of the viral load.

Chest CT scans at maximum 1 day before or after hospital admission were considered for image analysis at admission. For some patients, we obtained multiple CT scan data. For these patients, we compared the data collected during hospitalization with those obtained after hospital discharge (1–7 CT scans/patient).

### Imaging

All the CT images used in the study were acquired on one of our five multidetector CT scanners [Siemens Edge Plus (2), GE Revolution CT (1), and GE Brightspeed (2)]. Since CT images were collected retrospectively, no standardized scan protocol was available over the complete dataset.

All the acquired CT scans were analyzed using the CE 0344 certified Class IIa medical device CAD4COVID-CT (Thirona, Nijmegen, Netherlands). This AI-based software package analyses the lungs and each of the individual lobes for automatic quantification of COVID-19-related pulmonary parenchymal involvement. The software uses state of the art deep learning and image normalization techniques to provide robust and repeatable quantitative information in CT scans acquired with varying scanner parameters, typically found in a clinical setting. The analysis starts by identifying the lungs and each of the pulmonary lobes to provide their volumes. Within each of these areas of interest, emphysematous areas and COVID-19-related abnormality areas are identified and quantified. Emphysema is a pathological situation that worsens hypoxemia. Since hypoxemia is a critical factor in determining admission to the intensive care unit, it is important to distinguish whether the hypoxemia observed is due to emphysema or viral pneumonia.

This information is presented as the volume percentage of emphysema and volume percentage of the affected area for the whole lung and each of the pulmonary lobes. For each lobe, the percentage of the affected area (% AA) is used to calculate a severity score per lobe.

This lobar CT-SS is a categorization of the percentage of affected area defined as: 0 (affected area: 0%), 1 (affected area: 0.1–5.0%), 2 (affected area: 5.1–25.0%), 3 (affected area: 25.1–50.0%, 4 (affected area: 50.1–75.0%), and 5 (affected area: over 75.0%).

**FIGURE 1:**
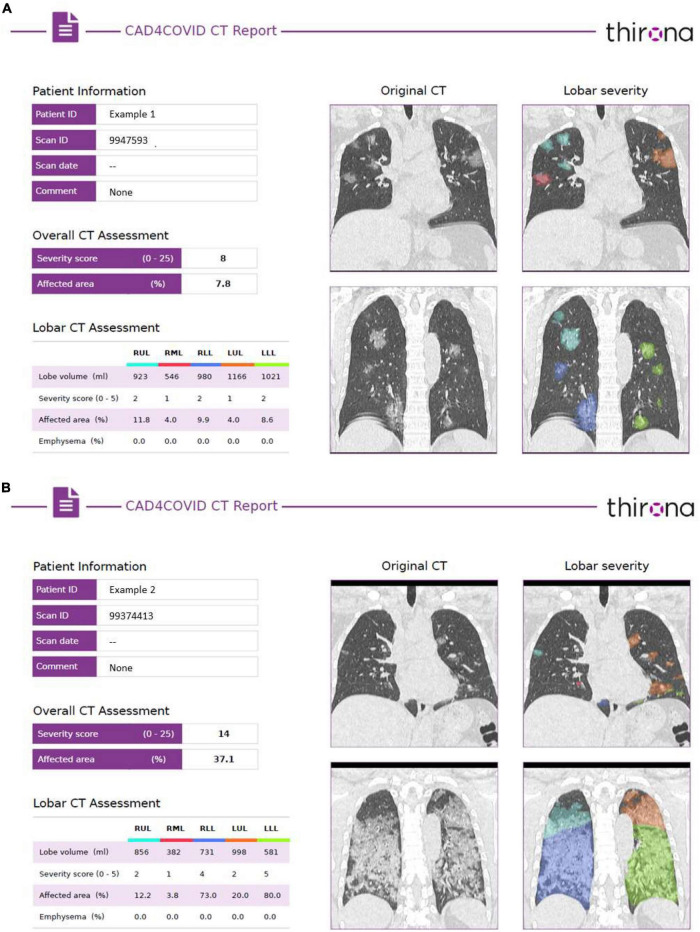
Example of the CAD4COVID-CT reports of two patients with COVID-19. **(A)** The left side of each report provides the quantitative assessment, including the lobar volume, the total and lobar CT severity scores and% affected area, and the lobar emphysema scores. **(B)** The right side of each report shows two coronal sections with a color-coated overlay of the identified affected areas, where each color represents a different lobe matching the colors indicated in the lobar CT assessment table.

The total CT-SS is the accumulation of each of the individual lobar scores. Two examples of CAD4COVID-CT report with all the quantitative information and an example of a coronal section of the CT scan are shown in Figure 1.

### Statistical methods

Quantitative variables were expressed by mean and SD, or median and P25 and P75 quartiles. Qualitative variables are presented using frequency tables (number and percentage).

The univariate linear regression models were used to study the relationship between the patient’s characteristics and CT-severity score (total CT-SS) and% affected area (% AA) at hospital admission.

The impact of the CAD4COVID-CT scores on the hospital length of stay (LOS) and the ICU length of stay was studied using the multiple linear regression models on the log-transformed lengths of stay. Results were presented as adjusted estimated coefficients ± SEs and *p*-values. The impact of the CAD4COVID-CT scores on the risk of ICU stay, the risk of ventilation, and the risk of in-hospital death was studied using the multiple logistic regression models. Results were expressed as adjusted odds ratios (ORs) and 95% CI. All the multiple models were adjusted for age, gender, BMI, and wave.

Optimal total CT-SS cutoff points to predict ICU admission and the need for mechanical ventilation were calculated on the data of wave 2 using Youden’s index method. The predictive models based on the data of W2 were recommended for optimal cutoff point estimation, since the risk of the considered outcomes had changed between W1 and W2. As the number of cases increased significantly during W2, it was also imperative to adapt the ICU admission procedure, to preserve resources for the most severe cases.

Generalized linear mixed models (GLMMs) were used to analyze the evolution of the CAD4COVID-CT scores with time during and after the hospital stay.

The results were considered significant at the 5% uncertainty level (*p* < 0.05). Statistical analyses were performed on all the available data and the missing data were not replaced. Calculations were done using SAS software (version 9.4) and graphics with R software (version 3.6.1).

## Results

### Patients characteristics

A total of 476 patients with COVID-19 hospitalized at the CHU Hospital of Liège between 13 March 2020 and 18 April 2021 were included in the study. Patients’ characteristics are given in Table 1.

**TABLE 1:** Patients’ characteristics.

Characteristics	Results Mean ± SD, Median (P25 – P75), or N (%)
Age (years)	67.3 ± 14.7
Gender, Men	311 (65.3)
BMI (kg/m^2^)	28.5 ± 6.3
Chronic kidney disease	52 (14.2)
Diabetes	204 (48.7)
Arterial hypertension	293 (64.5)
Cardio-vascular disease	138 (38.3)
Chronic respiratory disease	114 (27.8)
Immunosuppressive therapy	22 (6.0)
Obesity	153 (37.1)
Oncological condition	63 (13.2)
Wave 1	229 (48.1)
Inter Wave 1-Wave 2	8 (1.7)
Wave 2	226 (47.5)
Wave 3	13 (2.7)
Hospital LOS (days)	11 (7 – 19)
ICU	240 (50.4)
Time between hospital admission and ICU admission (days)	2 (1 – 3)
ICU LOS (days)	8 (4 – 18)
Mechanical Ventilation	134 (28.1)
Dialysis	22 (4.6)
In-hospital death	144 (30.2)

Within our cohort, we identified that 37.1% of them were suffering from obesity, defined as a body mass index (BMI) above 30 kg/m^2^.

The relationship between the characteristics of the patients and total CT-SS and% AA at hospital admission was calculated. They were significantly correlated with BMI and obesity (*p* < 0.001). We observed that older patients had lower% AA (*p* < 0.0001) and CT-SS (*p* < 0.001) at admission. Patients with the oncological conditions had lower% AA (*p* < 0.01) and CT-SS (*p* < 0.05) at admission. Neither CT-SS at admission nor% AA was related to gender (Supplementary Data, Tables 1, 2).

**TABLE 2:** CT scans analyses by Thirona (*n* = 476).

	Mean ± SD	Median (p25-p75)	Extremes
Volume (mL)	3411 ± 1184	3386 (2637 – 4105)	1340; 8578
Emphysema score (%)	0.74 ± 2.8	0.024 (0.0001 – 0.22)	0.00; 31.7
% AA	26.1 ± 22.4	19.0 (6.3 – 42.2)	0.00; 84.1
Total CT-SS	11.4 ± 6.0	11 (7 – 16)	0; 25

We also analyzed the relationship between BMI, age, wave, and the severity of hospitalization indicators (Supplementary Data, Table 3). Patients with higher BMI had higher LOS (*p* < 0.05), higher risk of ICU admission (*p* < 0.05), higher ICU LOS (*p* < 0.05), and higher risk of mechanical ventilation (*p* < 0.05).

**TABLE 3:** Relationship between CT scan analysis and hospitalization severity.

	Length of stay		Risk of ICU admission		ICU length of stay		Risk of mechanical ventilation		Risk of in-hospital death	
	Coef ± SE	*P*-value	OR (95% CI)	*P*-value	Coef ± SE	*P*-value	OR (95% CI)	*P*-value	OR (95% CI)	*P*-value
CT-SS	0.025 ± 0.0071	<0.001	1.3 [1.2; 1.3]	<0.0001	0.017 ± 0.012	0.16	1.2 [1.1; 1.2]	<0.0001	1.0 [1.002; 1.1]	<0.05
% AA	0.0060 ± 0.0019	<0.01	1.1 [1.1; 1.1]	<0.0001	0.0036 ± 0.0029	0.22	1.0 [1.03; 1.05]	<0.0001	1.0 [1.002; 1.02]	<0.05

All the models were adjusted for age, gender, BMI, and wave.

Hospital and ICU LOS were lower when diagnosis occurred after wave 1 (*p* < 0.05 and *p* < 0.0001, respectively).

Older patients have a higher risk of ICU admission (*p* < 0.01) and a higher risk of death during their hospital stay (*p* < 0.0001).

Gender was not related to the risk of hospitalization severity in this study for none of the severity parameters measured.

### CT Scans at admission: CAD4COVID-CT analysis

The 476 CT scans at a maximum 1 day before or after hospital admission were analyzed. A corresponding severity score was assigned to each scan (Table 2), depending on% AA.

We also analyzed CT-SS and% AA for each lobe. We observed that the most affected lobes were the lower ones for both the analyses (Supplementary Table 4).

### Relationship between CAD4COVID-CT analysis and hospitalization severity indicators

An increased total CT-SS at admission, as well as the% AA, was closely associated with a higher risk of prolonged hospital LOS, ICU admission, mechanical ventilation, or in-hospital death. Of note, there was no specific correlation with ICI LOS (Table 3).

When we considered the scores obtained from the individual lobes, we observed that the highest order of% AA and CT-SS was reached in the left and right lower lobes. However, the association with patients’ outcomes was in the same range of statistical significance for LOS, risk of ICU admission, ICU LOS, and risk of mechanical ventilation, except for the risk of in-hospital death, where the *p*-values were > 0.05 (Supplementary Table 5).

### Specificity and sensitivity of CAD4COVID-CT as a predictive tool of intensive care unit admission and mechanical ventilation risks

The predictive models based on the data of wave 2 (W2) were recommended for optimal cutoff point estimation since the risk of the considered outcomes had changed between waves 1 (W1) and W2. As the number of cases increased significantly during W2, it was also imperative to adapt the ICU admission procedure, to preserve resources for the most severe cases.

**FIGURE 2:**
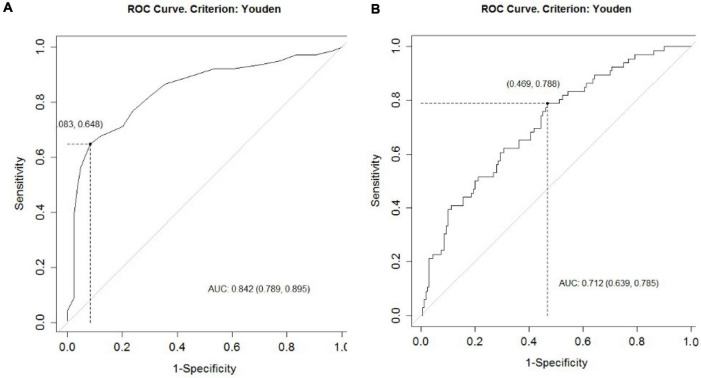
The ROC curve predicts ICU admission based on the initial CAD4COVID-CT evaluation. The AUC (95% CI) and optimal cutoff determination for the CT-SS to predict **(A)** risk of ICU admission and **(B)** risk of mechanical ventilation.

We calculated Youden’s index to maximize specificity and sensitivity, for the CT-SS for the 226 patients who experienced COVID-19 during W2 (Table 4). Results showed a CT-SS cutoff of 14 to predict the risk of ICU admission and 16 for mechanical ventilation. Figure 2 shows the area under the curve (AUC) (95% CI) and optimal cutoff determination for CT-SS to predict the risk of ICU admission (A) and risk of mechanical ventilation (B).

**TABLE 4:** The CT-SS cutoff points (wave 2, *n* = 226).

	ICU admission	Mechanical ventilation
AUC (95%CI)	0.84 (0.79; 0.90)	0.71 (0.63; 0.78)
Optimal cut-off point	14	16

External validation should be necessary for cutoff validation. Even if the W1 group was not representative of the current situation of the patients (most of the outcomes were improved between W1 and W2) to test the cutoff values, we nevertheless applied the cutoffs to the W1 group as internal validation (*n* patients = 229).

The CT-SS cutoff of 14 to predict the risk of ICU admission led to a sensitivity of 87% (95% CI: 81 to 92%) and a specificity of 58% (95% CI: 47 to 68%); the CT-SS cutoff of 16 to predict the risk of mechanical ventilation led to a sensitivity of 88% (95% CI: 83 to 93%) and a specificity of 48% (95%CI: 35 to 60%). These results are very similar to those obtained with W2 patients.

### Computed tomography scan evolution over time

**FIGURE 3:**
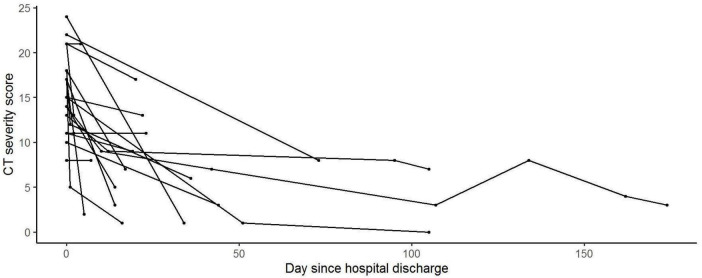
CT-SS evolution after hospital discharge. Generalized linear mixed models (GLMMS) were used to analyze the evolution of the CT’s Thirona scores with time during and after the hospital stay.

**FIGURE 4:**
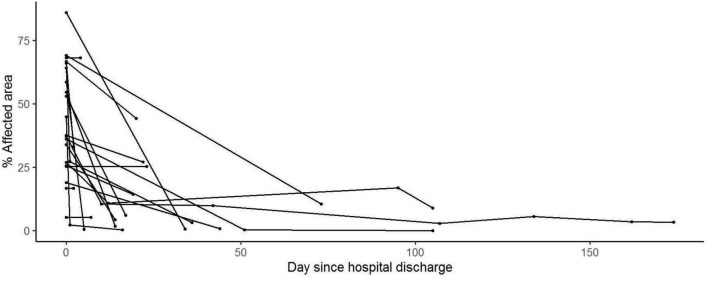
% Affected area evolution after hospital discharge. Generalized linear mixed models (GLMM) were used to analyze the evolution of the CT’s Thirona scores with time during and after the hospital stay.

Of the 476 patients, 84 patients had repeated chest CT scans during hospitalization and follow-up period. Figures 3, 4 show the evolution of total CT-SS and% AA after hospital discharge (GLMM model). Value considered at hospital discharge (time = 0 in the figure) was the maximum CT-SS or% AA during the hospital stay (*n* = 20 patients with at least one CT scan after hospital discharge).

We observed that both the measured values are decreasing after discharge, with a significant evolution over time (*p* < 0.001 and *p* < 0.01, respectively).

## Discussion

In our study, we showed that CAD4COVID-CT was able to help in the risk stratification of patients suffering from acute COVID-19 infection, based on chest CT images. Both the total CT-SS and% AA were able to predict hospital LOS, ICU admission risk, risk of mechanical ventilation, and in-hospital death. This means that AI can be used with great efficiency to predict the risk of worsening the patient’s condition, thus allowing for better management of patient flow in the hospital.

Our analysis showed that this CT scan image analysis tool can be of interest to better stratify the risk of ICU for patients acutely infected with COVID-19. This approach can help in the global management of patients in an in-hospital setup.

We also analyzed the relationship between patients’ characteristics and the% AA or total CT-SS. Patients with higher BMI had higher% AA and CT-SS at admission, and the severity indicators were all significantly related to this condition. The mean BMI of our cohort (28.5) was higher than in the whole Belgian population (being 25.5) (20). About half of the Belgian population is overweight, making it one of the top five factors associated with mortality in our country. The Belgian adult population has an obesity rate of 15.9% (20), while our cohort showed a 37.1% obesity rate. This is consistent with much-published data associating age and BMI with the risk of severe COVID-19 (2, 21–24).

In a counterintuitive way, we observed that older patients had lower% AA and CT-SS at admission, while they died more frequently. This could be explained by the comorbidities found in this population, which can increase the global risk of experiencing complications associated with COVID-19 infection. Similarly, patients with oncological conditions had lower% AA and CT-SS at admission, which can also be explained by non-COVID-19-related associated complications.

We showed that hospital LOS, ICU admission risk, and ICU LOS were lower when diagnosis occurred after W1. At this time, more effective care had been setup in our hospital, including early treatment with dexamethasone and remdesivir, as well as high-flow nasal oxygen therapy. This resulted in a decrease in ICU admission risk, ICU length of stay, and mechanical ventilation risk, among other indicators (25).

The secondary aim of this study was to determine whether CAD4COVID-CT was able to help in disease monitoring and follow-up. Some patients benefited from a long-term follow-up, with several CT scans after discharge from the hospital (5, 26). For those patients, we showed that the total CT-SS and% AA decreased over time. This finding was consecutively strongly correlated with health improvement as expected (27). Importantly, chest CT should not be considered as a follow-up tool for COVID-19 infection due to radiation-associated risk and the lack of rationale for systematic follow-up.

CAD4COVID-CT provides two main AI-based scores: (1) the percentage of the affected area (% AA) and (2) the categorical CT severity score derived from the affected area (total CT-SS). Although these two scores are highly related to each other, both provide a specific value for the CT assessment. The% AA is the most precise measure, which is calculated at the voxel level. This allows for an exact delineation and quantification of the percentage of affected lung tissue. The CT-SS score is derived from the% AA similar to the CO-RADS, in which certain cutoff points are used to make severity categories. Evidently, by categorizing a continuous variable, precision information is lost. However, categorical scores allow for a direct comparison to a visual assessment, as it mimics how a human would score disease severity, where true quantification for humans is virtually impossible. Therefore, using AI for precise quantification of COVID-19-affected lung areas gives reliable results, and a good correlation with disease severity, while scoring may reassure clinicians and radiologists, who are used to visually assigning severity scores when reviewing CT scans (13). An additional advantage of using AI-based quantification is in statistical analysis and risk stratification. Moreover, an AI-based algorithm provides consistent and objective output, while allowing avoiding potential discrepancies in inter- and intraobserver variability.

An important aspect of CAD4COVID-CT is that the entire analysis was designed to handle the considerable amount of CT scan variability, typically encountered in clinical practice and especially during the COVID-19 pandemic. Different sources of CT scan variability (such as differences in scanner manufacture, reconstruction kernels, and dose levels) can have a substantial impact on the quantitative score if not properly mitigated during algorithm development. This may lead to poor clinical correlations and conflicting longitudinal assessments. The design of CAD4COVID-CT allows the algorithm to deal with CT scan variability in two main ways. First, the AI-based algorithms were trained with a well-balanced set of CTs coming from various sources. This allowed the algorithms to learn from CT scan variations that they would likely encounter in a clinical setting. Second, CT scan normalization techniques are used to standardize each CT before the scan is presented to the AI algorithms. This procedure greatly reduces the inherent variability between scans and allows the AI algorithms to provide consistent results in a clinical setting.

A limitation of the CAD4COVID-CT analysis is the lack of separation of different textures within the identified affected areas. The AI-based algorithm was trained to identify COVID-19-related abnormalities as a single class, meaning that both the ground-glass opacities and consolidations are combined into the% AA and CT-SS scores. Although this approach follows the severity scoring of the CO-RADS, separating ground-glass opacities and consolidation could provide additional clinically relevant information on disease severity and prognosis. Furthermore, the current quantification is lobar-based, allowing a quantitative assessment of the lobar disease distribution. With only the lobar information, quantification of the ventral vs. dorsal disease distribution is not possible, while this information may be clinically relevant in patients that require mechanical ventilation. However, since CAD4COVID-CT is identifying the abnormalities on a voxel level, and the relationship between the voxels and the lung and lobar boundaries is known, this information could be extracted and added as an additional feature.

Another limitation of this study comes from the fact that this is a retrospective study performed in a single center. It should also be validated on external data.

## Conclusion

In conclusion, our study showed that the CAD4COVID-CT AI-based quantification of lung injury in COVID-19 infection was highly correlated with major clinical indicators and helped to predict ICU admission and the risk of mechanical ventilation. This method can be used as a clinical decision support system for patients’ triage, to better manage the intrahospital flow, and to guide the indicated therapy promptly. Further clinical studies for validation are needed to confirm the added value of this model depending on the variant modification over time.

## Data availability statement

The datasets generated or analyzed during this current study are not publicly available because these data are considered sensitive, but are available from the corresponding author on reasonable request.

## Ethics statement

The studies involving human participants were reviewed and approved by the Ethics Committee of the University Hospital of Liege (Comité d’éthique hospitalo-universitaire de Liège) reviewed the study and approved it (Reference: 2022/21). Written informed consent for participation was not required for this study in accordance with the national legislation and the institutional requirements.

## Author contributions

JG, RL, and BL conceived the study. RL, BM, and BL designed the experiments. MH and NM analyzed the results. JG, MT, A-NF, PM, A-FR, PC, and BM conducted the experiments and acquired the data. BE acquired the funding. J-PC provided the Thirona software. JG, NM, MW, A-FR, J-PC, and BL wrote the manuscript. All authors have read and approved the final version of the manuscript.
