# Supplementary material for: Automatized lung disease quantification in patients with COVID-19 as a predictive tool to assess hospitalization severity
Source: Front Med (Lausanne). 2022 Aug 29;9:930055. doi: 10.3389/fmed.2022.930055 (PMC9465374; doi:10.3389/fmed.2022.930055)
Supplement: Supplementary file 1 [file Data_Sheet_1.pdf]

Automatized lung disease quantification in COVID-19 patients as a predictive tool to assess hospitalization severity

Supplementary data

Clinical and demographic characteristics

For each patient, the following data are available:

- Age (years)
- Gender (0=F, =M)
- Length (cm)
- Weight (kg)
- BMI (kg/m²)
- Comorbidities
- Date of first positive PCR
- Admission date (= PCR date unless PCR date is prior to hospital admission date. In this case, Day0 = Hospitalization date).
- Wave of the epidemic of Covid-19 (source = Sciensano, Annex 1)
    - first wave from March 1 to June 22, 2020 (W1)
    - inter wave period from June 23, 2020 to Augustus 31, 2020 (Inter W1-W2)
    - second wave from September 1,2020 to February 13, 2021 (W2)
    - third wave started on February 14, 2021 (W3)
- Discharge date (= Discharge date or transfer date to revalidation)
- Length of stay (days)
- Total ICU length of stay (days)
- Number of days between hospital admission and ICU admission
- Symptoms at admission
- Ventilation (1=Yes, 0=No)
- Dialysis (1=Yes, 0=No)
- Issue (0=discharged alive, 1=death)
- CT-scan date
- Number of days between hospital admission and CT scan
- CT severity score: CT severity score for each lobe (0 to 5).
- Total CT severity score = Sum of scores obtained from each lobe = principal outcome (0 to 25). Higher score = higher severity.
- Total lungs volume (mL)
- % Affected area = % of total affected lung parenchyma obtained by sum of each lobe % affected area
- % Emphysema score: % of emphysema of affected area for the whole lung

Impact of patients' characteristics on CT scans at admission

We studied the relation between the characteristics of the patients and CT-SS or % AA at hospital admission using univariate linear regression models. Univariate linear regression models were used to study the relation between the characteristics of the patients and CT-SS or % AA at hospital admission (Tables 5 and 6).
We observed that older patients had lower % AA ($p<0.0001$) and CT-SS ($p=0.0002$) at admission. Obese patients and patients with higher BMI had higher % AA ($p<0.0001$) and CT-SS ($p=0.0001$) at admission. Patients with oncological condition were also exhibiting lower

% AA (p=0.0082) and lower CT-SS (p=0.018) at admission. Neither CT-SS at admission nor % AA was related to gender.

Supplementary Table 1. Relation between patients' characteristics and CT-SS at admission (N=476)

Patient's characteristics	Coef. ± SE	p-value
Age (years)	-0.070 ± 1.3	0.0002
Gender (1=Men)	0.96 ± 0.58	0.096
BMI (kg/m²)	0.20 ± 0.047	0.0001
Chronic renal failure	0.023 ± 0.87	0.98
Diabetes	0.75 ± 0.57	0.19
Arterial hypertension	0.12 ± 0.59	0.83
Cardio-vascular disease	-1.3 ± 0.78	0.11
Chronic respiratory disease	-0.49 ± 0.65	0.45
Immunosuppressive therapy	-2.4 ± 1.3	0.056
Obesity	2.3 ± 0.60	0.0001
Oncological condition	-1.9 ± 0.81	0.018

Supplementary Table 2. Relation between patients' characteristics and % AA at admission (N=476)

Patient’s characteristics	Coef. ± SE	p-value
Age (years)	-0.29 ± 0.069	<0.0001
Gender (1=Men)	3.4 ± 2.1	0.11
BMI (kg/m²)	0.79 ± 0.17	<0.0001
Chronic renal failure	-0.71 ± 3.3	0.83
Diabetes	3.1 ± 2.1	0.14
Arterial hypertension	0.57 ± 2.2	0.80
Cardio-vascular disease	-4.5 ± 3.1	0.15
Chronic respiratory disease	-1.2 ± 2.4	0.63
Immunosuppressive therapy	-7.0 ± 4.9	0.15
Obesity	9.4 ± 2.2	<0.0001
Oncological condition	-8.0 ± 3.0	0.0082

Supplementary Table 3. Relation between patient's characteristics and hospitalization severity indicators (Multiple linear regression model on log-transformed (ICU) or hospital LOS or multiple logistic regression model; models are adjusted for age, gender, BMI, and wave ; N = 410). LOS: length of stay.

	Hospital LOS		ICU admission risk		ICU LOS		Mechanical ventilation risk		In-hospital death risk	
	p value	Coef. ± SE	p value	OR [95%CI]	p value	Coef. ± SE	p value	OR [95%CI]	p value	OR [95%CI]
**Intercept**		1.7 ± 0.32				1.2 ± 0.54				
**Age (by decades)**	0.25	0.034 ± 0.030	**0.0008**	0.74 [0.62 ; 0.88]	0.23	0.063 ± 0.052	0.68	0.97 [0.81 ; 1.1]	**<0.0001**	1.7 [1.4 ; 2.1]
**Gender (Ref=Woman)**	0.90	0.010 ± 0.087	0.092	1.6 [0.93 ; 2.7]	0.82	-0.032 ± 0.14	0.33	1.3 [0.78 ; 2.1]	0.13	1.5 [0.89 ; 2.4]
**BMI (kg/m²)**	**0.045**	0.014 ± 0.007	**0.023**	1.0 [1.001 ; 1.1]	**0.011**	0.029 ± 0.011	**0.016**	1.1 [1.01 ; 1.1]	0.055	0.96 [0.92 ; 1.001]
**Wave (Ref=W1)**	**0.040**	-0.17 ± 0.083	**<0.0001**	3.4 [2.1 ; 5.7]	**<0.0001**	-0.65 ± 0.14	0.24	0.75 [0.47 ; 1.2]	0.59	1.1 [0.72 ; 1.8]
**Total CT severity score**	**0.0004**	0.025 ± 0.0071	**<0.0001**	1.3 [1.2 ; 1.3]	0.16	0.017 ± 0.012	**<0.0001**	1.2 [1.1 ; 1.2]	**0.040**	1.0 [1.002 ; 1.1]

Supplementary Table 4. Disease extension measured for each individual lobe.

	N non-missing	Mean ± SD	Median (p25-p75)	Extremes
		**CT severity score**		
Left upper lobe	476	1.9 ± 1.4	2 (1 – 3)	0 ; 5
Left lower lobe	476	2.7 ± 1.4	3 (2 – 4)	0 ; 5
Right upper lobe	476	2.1 ± 1.4	2 (1 – 3)	0 ; 5

Right lower lobe	476	2.8 ± 1.4	3 (2 – 4)	0 ; 5
Right middle lobe	476	1.8 ± 1.3	2 (1 – 3)	0 ; 5
Whole lung	476	11.4 ± 6.0	11 (7 – 16)	0 ; 25
		**Affected area (%)**		
Left upper lobe	476	20.6 ± 23.0	11.2 (1.3 ; 34.9)	0.00 ; 86.3
Left lower lobe	476	35.0 ± 28.8	61.5 (28.3 – 61.5)	0.00 ; 89.8
Right upper lobe	476	23.0 ± 24.2	14.2 (1.7 – 39.6)	0.00 ; 93.0
Right lower lobe	476	35.9 ± 27.6	31.6 (9.7 – 61.1)	0.00 ; 92.4
Right middle lobe	476	18.8 ± 23.1	7.5 (1.1 – 29.8)	0.00 ; 94.5
Whole lung	476	26.1 ± 22.4	19.0 (6.3 – 42.2)	0.00 ; 84.1

Supplementary Table 5. Association of CT scan analysis the with the patient outcomes for left and right lower lobes, compared to the whole lung.

	**Length of stay ***		**Risk of ICU admission §**		**ICU length of stay ***		**Risk of mechanical ventilation §**		**Risk of in-hospital death §**	
	Coef ± SE	p-value	OR (95% CI)	p-value	Coef ± SE	p-value	OR (95% CI)	p-value	OR (95% CI)	p-value
**CT-SS (Entire lung)**	0.025 ± 0.0071	0.0004	1.3 [1.2 ; 1.3]	<0.0001	0.017 ± 0.012	0.16	1.2 [1.1 ; 1.2]	<0.0001	1.0 [1.002 ; 1.1]	0.040
**% AA (Entire lung)**	0.0060 ± 0.0019	0.0018	1.1 [1.1 ; 1.1]	<0.0001	0.0036 ± 0.0029	0.22	1.0 [1.03 ; 1.05]	<0.0001	1.0 [1.002 ; 1.02]	0.023
**CT-SS (Left lower lobe)**	0.086 ± 0.030	0.0040	2.1 [1.8 ; 2.6]	<0.0001	0.055 ± 0.049	0.25	1.7 [1.4 ; 2.0]	<0.0001	1.1 [0.98 ; 1.4]	0.085
**% AA (Left lower lobe)**	0.0042 ± 0.0015	0.0044	1.0 [1.03 ; 1.05]	<0.0001	0.0022 ± 0.0023	0.34	1.0 [1.02 ; 10.3]	<0.0001	1.0 [1.00 ; 1.02]	0.062
**% AA (Rigth lower lobe)**	0.0052 ± 0.0015	0.0006	1.0 [1.03 ; 1.05]	<0.0001	0.0030 ± 0.0024	0.21	1.0 [1.02 ; 1.03]	<0.0001	1.0 [1.00 ; 1.02]	0.044

** Multiple linear regression models on log-transformed length of stay, § Multiple logistic regression models. All multiple models were adjusted for age, gender, BMI, and wave.*
